# High Neonatal Blood Iron Content Is Associated with the Risk of Childhood Type 1 Diabetes Mellitus

**DOI:** 10.3390/nu9111221

**Published:** 2017-11-06

**Authors:** Julie Nyholm Kyvsgaard, Anne Julie Overgaard, Steffen Ullitz Thorsen, Thomas Hesselhøj Hansen, Christian Bressen Pipper, Henrik Bindesbøl Mortensen, Flemming Pociot, Jannet Svensson

**Affiliations:** 1 Copenhagen Diabetes Research Center (CPH-DIRECT), Department of Paediatrics, Herlev University Hospital, 2730 Herlev, Denmark; Juliekyvs@hotmail.com (J.N.K.); Anne.julie.overgaard@regionh.dk (A.J.O.); s.u.thorsen@gmail.com (S.U.T.); henrik.bindesboel.mortensen@regionh.dk (H.B.M.); flemming.pociot.01@regionh.dk (F.P.); 2 Department of Plant and Environmental Sciences, Faculty of Science, University of Copenhagen, 2000 Frederiksberg, Denmark; thh@plen.ku.dk; 3 Section of Biostatistics, Department of Public Health, Faculty of Health and Medical Sciences, University of Copenhagen, 2099 Copenhagen, Denmark; pipper@sund.ku.dk

**Keywords:** diabetes mellitus, type 1, iron, embryonic and fetal development, pediatrics, newborn

## Abstract

(1) Background: Iron requirement increases during pregnancy and iron supplementation is therefore recommended in many countries. However, excessive iron intake may lead to destruction of pancreatic β-cells. Therefore, we aim to test if higher neonatal iron content in blood is associated with the risk of developing type 1 diabetes mellitus (T1D) in childhood; (2) Methods: A case-control study was conducted, including 199 children diagnosed with T1D before the age of 16 years from 1991 to 2005 and 199 controls matched on date of birth. Information on confounders was available in 181 cases and 154 controls. Iron was measured on a neonatal single dried blood spot sample and was analyzed by laser ablation inductively coupled plasma mass spectrometry. Multivariate logistic regression was used to evaluate if iron content in whole blood was associated with the risk of T1D; (3) Results: A doubling of iron content increased the odds of developing T1D more than two-fold (odds ratio (95% CI), 2.55 (1.04; 6.24)). Iron content increased with maternal age (*p* = 0.04) and girls had higher content than boys (*p* = 0.01); (4) Conclusions: Higher neonatal iron content associates to an increased risk of developing T1D before the age of 16 years. Iron supplementation during early childhood needs further investigation, including the causes of high iron in neonates.

## 1. Introduction

The incidence of childhood type 1 diabetes mellitus (T1D) is increasing, especially in Western countries [1,2], however the causes remain unknown. T1D is an immune-mediated disease where autoreactive T-cells are the major drivers in destruction of insulin-producing pancreatic β-cells [3]. When compared to earlier generations, the proportion of patients with high-risk human leukocyte antigen (HLA) alleles has not increased [4]. Therefore, it seems likely that environmental factors play an important part in the etiology of T1D. Studies suggest that T1D may already have its origin in fetal life [5], e.g., through modification of epigenetic processes in the fetus [6]. 

Iron, the most abundant metal in the body, is an essential trace element and has numerous functions. The most important is oxygen transportation bound to hemoglobin. Iron is also a key element in immune homeostasis, and studies have shown that iron deficiency and overload may affect T-cell counts and function [7,8].

Patients with late-onset T1D are reported to have an increased incidence of hereditary hemochromatosis [9]. Furthermore, in a study of 26 patients with homozygous β-thalassemia major, who had iron accumulation due to multiple blood transfusions, 50% had glucose intolerance and 19% had unspecified diabetes that was based on an oral glucose tolerance test [10]. Further, elevated transferrin saturation is found to be associated with an increased risk of both T1D and type 2 diabetes mellitus [11]. These studies suggest that high iron status may be a contributing factor in the pathogenesis of diabetes. Whether the driving factor is iron toxicity against the β-cell and/or a modulation of the immune system is unknown [12]

Iron deficiency and anemia are common conditions affecting nearly half of all pregnant women worldwide [13]. Iron requirement increases during pregnancy, which is why iron supplementation during pregnancy is recommended [13]. However, in Western countries, the need for iron supplementation may not always be necessary due to an optimal nutritional status. Further, the supplement dose taken can be above the recommended amount [14]. In fact, one study showed that 37% of Danish pregnant women had an iron supplementation intake above the recommended 50–70 mg per day [14].

The mechanisms by which the placental barrier regulates the maternal-fetal exchange of substances are relatively unknown. The placenta has the ability to up-regulate fetal iron in terms of maternal iron deficiency [15], but there is limited knowledge on the placental regulation regarding maternal iron overload.

We hypothesized that higher neonatal iron content in blood is associated with an increased risk of the β-cell injury, and therefore an increased risk of developing T1D in childhood. Therefore, the aim of the study was to investigate whether the neonatal iron content in blood is associated with the risk of developing T1D before the age of 16 years.

## 2. Materials and Methods

### 2.1. Data Source and Study Design

A case-control design was used. Two hundred children clinically diagnosed with T1D (cases), born in Denmark between January 1991 and November 1998, were randomly selected from the Danish Childhood Diabetes Registry (DanDiabKids). 

Power calculation: No data for Fe^56^ was available; therefore we used data from a study of neonates documenting a mean value of 94.9 µg/dL and standard deviation score of 7.2 µg/dL [16]. Using these data we estimated a power of 97.5% to detect an odds ratio (OR) of 1.3 or 0.77, when including 199 cases and controls with a significance level of 5%.

Inclusion criteria were: age at diagnosis <16 years and an available neonatal dried blood spot screening card (NDBS) present in the Danish Newborn Screening Biobank [17]. One case/control pair was excluded prior to iron analysis because of the misplacement of NDBSs.

One control for each case was selected by using consecutive screening numbers (equivalent to date of birth). Cases and controls were all identified as part of a study on T1D and early risk factors [18]. The study period (1991–1998) was selected due to the usage of the same type of filter paper during this period, thereby eliminating the risk of including filter paper with different iron-binding capacity. The diagnosis of T1D was classified according to World Health Organization criteria.

### 2.2. Sample Collection

The Danish Newborn Screening Program offers neonatal screening of several diseases to all of the children born in Denmark. The coverage is close to 100% [19]. Capillary blood samples were taken from the newborn’s heel, 5–7 days after birth, and immediately transferred to filter paper; the NDBSs. The material are stored in the Danish Newborn Screening Biobank at −25 °C/(−13 °F) [17]. One punch containing ~3.4 µL of dried capillary blood, from each participant, was used for iron analysis.

### 2.3. Analysis of Iron and HLA-DQB1 on NDBSs

The samples were analyzed for iron (^56^Fe) using laser ablation inductively coupled plasma mass spectrometry (LA-ICP-MS). The samples were also analyzed for potassium (^39^K) in the same run to control for blood volume on the NDBS and the hematocrit value in the newborn [19]. All of the samples from the cases and controls were analyzed in the same run to minimize operational variation. 

Filter paper samples containing Seronorm™ Trace Elements Whole Blood L-2 (SERO AS) were used as reference samples. Furthermore, empty filter paper samples were also analyzed to control for any contamination from the filter paper. The reproducibility between the 31 identical standard samples, in terms of coefficient of variation, was 19.3%.

LA-ICP-MS is a fast, multi-elemental technique, and as such, it was possible to measure 12 consecutive points on each punch, allowing for a better estimate of the iron content [20,21]. LA-ICP-MS measures the ion intensity in counts per second, which is proportional to the metal concentration. As we were measuring iron in capillary whole blood and divided with potassium the result is a ratio of the total content of iron ions in relation to the total content of potassium ions, therefore the results is not a concentration per µL of blood but in units. However, since we were looking at the differences between the samples, this does not affect the ability of the analysis to detect differences in whole blood iron content between cases and controls, and thereby the influence of iron content on subsequent risk of developing T1D. 

Genotyping for T1D associated HLA-DQB1 alleles was performed using DELFIA^®^ (PerkinElmer, Finland), which is a combination of polymerase chain reaction and time-resolved fluorometry, as described in detail elsewhere [18].

### 2.4. Descriptive Data

From relevant databases, i.e., DanDiabKids, the Newborn Screening Biobank [17], the National Patient Registry, and the Danish Birth Registry, the following demographic and anthropometric data on cases and controls were retrieved: sex, date of birth, maternal age at delivery, maternal diabetes diagnosis, birth weight, birth length, gestational age, and age at diagnosis (for cases). In addition, 89% (353/398) of the included individuals were HLA-DQB1 genotyped in a previous study [18]. More specifically, HLA-DQB1 genotyping was not possible in 16 cases and 29 controls due to blood sample quantity or quality of the NDBS. HLA-DQB1 genotypes were divided into high, moderate, and low/protective risk categories (HLA risk), based on international consensus [18,22] (detailed in Table 1). Season of birth was coded in the following manner: spring (March through May), summer (June through August), autumn (September through November), and winter (December through February). Birth year was divided in three equally sized periods (1991–1992, 1993–1994, and 1995–1998).

### 2.5. Statistical Analyses

For each individual, there were 12 simultaneous measures of iron and potassium content. To control for differences in blood volume and hematocrit concentration on each NDBS, iron was divided by potassium to normalize the iron content [19]. Contamination was thought to be the cause of two extreme outliers and these were deleted before a mean value was calculated for further analysis. 

The differences in birth variables between case and control populations were evaluated by the Welch two-sample *t*-tests, Wilcoxon rank-sum test or chi-square depending on distribution. The distribution of continuous variables was presented as mean and standard deviation (SD), or median and interquartile range, depending on the normality distribution assessment. The distribution of categorical variables was presented as frequencies. The iron measure is arbitrary, therefore iron was log2 transformed to calculate the OR for developing T1D associated with a doubling in iron content as the explanatory variable. Further, we tested iron in quartiles calculated based on the total sample. The missing data were judged to be completely at random and therefore a complete case analysis was performed. For our primary analyses, we used logistic regression with case-control status as the outcome variable and log-transformed iron values as the main exposure variable that was adjusted for the matching variables season and year of birth. The association between T1D and the other variables was likewise adjusted for season and year of birth. The following possible confounders were included: sex, maternal age at delivery, maternal diabetes diagnosis, birth weight, gestational age, and HLA risk. Birth weight and length are both measures of fetal growth and due to possible multicollinearity (*r* = 0.82, *p* < 0.0001), only birth weight was included in the adjusted regression. The association between the possible confounders and iron content was evaluated by one-way analysis of variance (ANOVA) or linear regression. All of the *P*-values were evaluated at a 5% significance level. 

SAS version 9.4 was used for all the statistical analyses. The study was approved by the Danish National Committee on Health Research Ethics (H-2-2014-007). According to Danish law, anonymous registry studies do not require further informed consent. 

## 3. Results

### 3.1. Basic Characteristics

**Figure 1:**
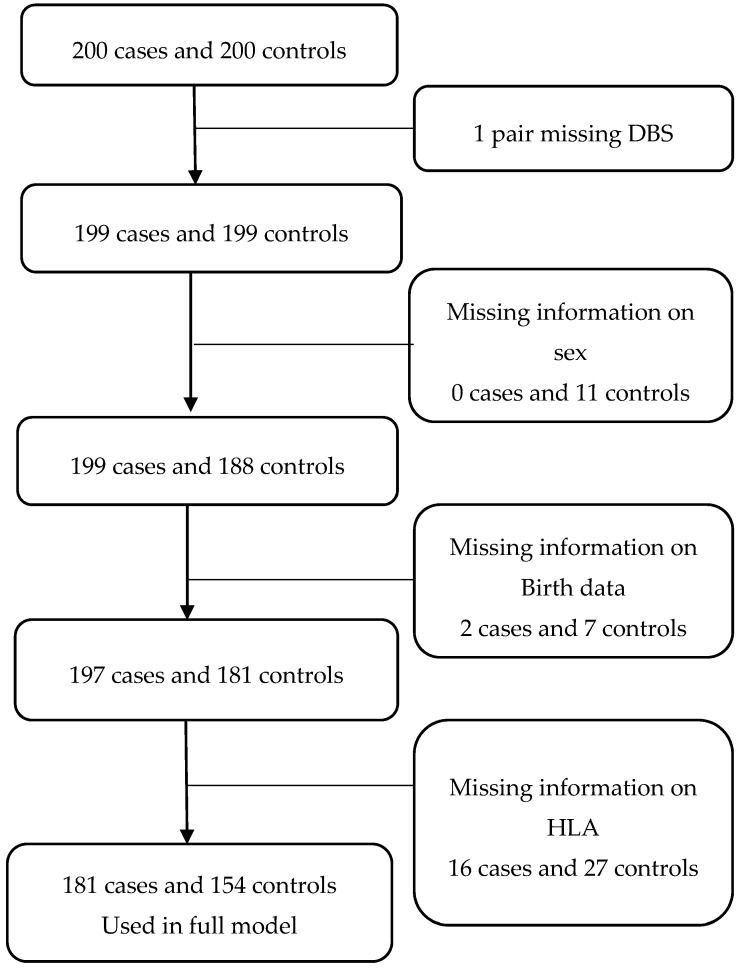
Flowchart illustrating patients participating in the study cohort.

Iron values were measured for 199 cases and 199 controls. Of these participants, 181 cases and 154 controls had information on all of the variables (inclusion and exclusions are presented in Figure 1). Overall, mean iron content was 1.80 (0.30) units for the 199 cases and 1.74 (0.39) units the 199 controls. 

Other characteristics of cases and controls are seen in Table 1. Notably, cases and controls were statistically comparable for sex, gestational age, birth length, birth weight, and maternal age at delivery. They differed according to HLA risk (Table 1). Maternal diabetes diagnosis was not included in the analysis as only one mother had this diagnosis.

To estimate the association between iron and the selected confounders, iron content was tested against each variable individually using one-way ANOVA or linear regression. Iron did not differ with season (*p* = 0.36), birth weight (*p* = 0.38), birth length (*p* = 0.07), and HLA risk (*p* = 0.68). Iron content was significantly lower in boys than in girls (beta-coefficient (95% confidence interval (CI)), −0.09 (−0.16; −0.02 units)) (*p* = 0.01) and increased per 1 year in maternal age at delivery (beta-coefficient (95% CI), 0.078 (0.003; 0.15 units)) (*p* = 0.04). By contrast, the association with birth year was only borderline significant in 1991–1992 (beta-coefficient (95% CI), −0.079 (−0.167; 0.009 units)); in 1993–1994 (beta-coefficient (95% CI), 0.017 (−0.070; 0.104 units)) relative to 1995–1998 (*p* = 0.053).

### 3.2. Iron and T1D Risk

The logistic regression model of iron content on T1D risk, also adjusted for birth year and season, resulted in a two-fold increase of developing T1D for each doubling of iron content (OR (95% CI), 2.07 (1.07; 4.00)) (*p* = 0.030). This association became even stronger after adjusting for possible confounders (sex, maternal age at delivery, birth weight, gestational age, and HLA risk) (OR (95% CI), 2.55 (1.04; 6.24)) (*p* = 0.041) (Table 2). 

When iron was divided in quartiles we found a significant lower risk of developing T1D in those belonging to the lowest quartile compared to highest quartile (*p* = 0.04).

## 4. Discussion

To our knowledge, this is the first study to investigate and demonstrate a positive association between neonatal iron content in blood and the risk of developing T1D before the age of 16 years. Furthermore, iron content was significantly lower in boys than in girls, and increased with maternal age.

The relationship between diabetes and iron status in children have only been scarcely studied. A retrospective study on the intake of iron in early infancy found a similar doubling in OR, for developing T1D, for every SD increase in iron intake [23]. The dietary intake was based on self-reporting questionnaires with few participants in the low-iron-formula group, therefore, the results could be biased because of a beneficial effect of breast milk on the risk of T1D [24]. A few studies conducted in adult populations have investigated markers of iron metabolism and concentrations in patients with T1D [25,26]. A study of 39 patients with T1D patients and 100 controls, found that patients had lower total iron binding capacity, lower transferrin, and higher haptoglobin levels, but found no sign of iron overload [25]. In 192 patients with long-term T1D and 59 healthy controls, no difference in plasma iron was found [26]. In contrast, others have found a doubling of the OR for developing T1D in those with a transferrin saturation above 50% (a measure of iron overload) [11].

The link between iron and diabetes is also indicated by the higher prevalence of diabetes in diseases with an iron overload. Mutations in the High Iron Fe (HFE) genes (C282Y or H63D), less frequent mutations in the genes coding for hepcidin, ferroportin, transferrin receptor 2 or hemojuvelin (HFE2) are causes of hemochromatosis, which are characterized by the increased absorption of iron [27,28]. The accumulation of iron leads to a diabetes that are characterized primarily of insulin deficiency, but also insulin resistance [12]. The β-cell failure after iron accumulation is in animal models shown to be secondary to oxidative stress that is caused by free radicals from the Fenton reaction [29]. 

Ferroportin regulates the iron absorption from the gut, and the number of ferroportin exporters is regulated by hepcidin. In hepcidin, knockout mice pancreatitis develops spontaneously [30]. Bone morphogenetic protein-6 (BMP-6) is a signaling molecule involved in iron homeostasis. In BMP-6 knockout mice, the islet of Langerhans are decreased in numbers [29] and glycaemia is reduced after the infusion of BMP-6 [31]. Overexpression of metal-ion transporters, such as the natural resistance-associated macrophage protein-1 (NRAMP1)/Slc11a1 [32], the divalent-metal transporter 1 (DMT1), also called NRAMP2/Slc11a2 [33], and ZRT/IRT-like protein 14 (ZIP14, transporting unbound iron into the cell) [34] are associated with increased iron uptake in different cell-lines (including dendritic cells, macrophages, and β-cells), and the development of T1D. Furthermore, iron is also involved in autoimmune processes, e.g., it functions as a catalyst in the formation of cryptic epitopes reconcilable for autoantigens [8]. In addition, iron has the ability to modify glutamic acid decarboxylase, a major β-cell autoantigen [35]. 

The iron concentration may also influence infections and gut microbiota since all microbes need iron for survival and optimal virulence [36]. Low iron concentrations work as a defense mechanism against infections; on the contrary, high iron inhibits the macrophages’ ability to kill intracellular pathogens. The role of infections as factors of T1D is not clear, but recent studies indicate a link between gut microbiota and T1D [37]. Taken together, these pieces of evidence indicate a role of iron in T1D etiology. 

We found a tendency towards an increase in neonatal iron content in blood in the children born in later generations. This might be explained by a heighten public health awareness during this time period i.e., resulting in an increase in mineral/vitamin supplement intake in pregnant Danish women. A higher iron concentration in girls as compared to boys has been documented previously [38], and a higher concentration in older pregnant women is in line with current knowledge about older pregnant women, they are more likely to use iron supplementation [14]. Though, a higher iron concentration in pregnant women does not necessary lead to higher levels in the newborn child, since the correlation between maternal and fetus levels of ferritin were visible only at lower levels of ferritin [15].

Our study has several strengths: (i) We included a relatively large number of participants; (ii) We used clinically validated cases and our study is population-based; and, (iii) Multiple possible confounders were taken into account. Some limitations also warrants attention: (i) We had no genetic data on key iron metabolism genes e.g., HFE, HJV, BMP6, Slc39A14, Slc11a1, and Slc11a2 [27,34], but our study was not constructed size-wise to elucidate gene-gene and gene-iron interactions; and (ii) The optimal method for quantifying iron status have been widely discussed [39]. Iron metabolism includes several steps, many of which involve known acute phase reactants, such as transferrin and ferritin. Ferritin is the most sensitive marker of iron deficiency and probably also of iron overload [40]. We were not able to measure transferrin, ferritin, or other aspects of iron metabolism separately in the NDBS. About 60–65% of iron in the body is in the hemoglobin, whereas only 0.1% is circulating in the blood bound to transferrin. Earlier research has not assessed whether the problem with iron overload, in relation to diabetes, stems from excess unbound blood iron, increased intracellular iron, or abundant body storage. We measured total ^56^Fe in capillary whole blood using LA-ICP-MS. LA-ICP-MS may not be a common way to measure iron in blood, but: (i) It is widely used for measuring several trace elements in biological samples [41]; (ii) It is an ideal method when the biological material consists of small amounts of dried blood; and, (iii) Quantifies whole blood iron status. Further, it has been proven to be valid in previous studies [20,21]. The fact that we confirm previous results found using more well-established methods, such as higher values in girls when compared to boys and the increasing levels with maternal age can be seen as a support for our method. Accumulation of iron granules in the pancreas and heart are seen with normal circulating concentrations of serum iron and transferrin in animals [29], indicating that an iron overload may be present and cause β-cell damage, without being detectable in the bloodstream. This supports our choice of method where total iron is measured. 

## 5. Conclusions

As the first study using direct neonatal iron content we report an association with T1D before the age of 16 years. Further research is obviously needed to prove causality, and to ensure that our results are not due to residual confounding e.g., by using an established unique cohort comprised of children that are genetically at risk of T1D. Further, causes of high neonatal iron such as genetic profile and correlation with maternal iron status needs further exploration before any change in guidelines should be made. 

## Figures and Tables

**Table 1:** Characteristics of study participants.

Variables (Median/Range)	Cases (*n* = 198)	Controls (*n* = 188)	*p* Value	Cases (*n* = 181)	Controls (*n* = 154)	*p*-Value	Missing Data (*n*/% of Total)
Sex							
Males (*n*/% of total)	96 (48.2)	92 (46.5)	0.81	87 (48.1)	78 (50.7)	0.81	11/2.8
Gestational age, weeks (median/range)	40 (31–43)	40 (27–43)	0.08	40 (31–43)	40 (28–43)	0.22	17/4.3
Birth length, cm (median/range)	52 (42–58)	52 (35–58)	0.30	52 (42–57)	52 (35–58)	0.18	21/5.3
Birth weight, kg (median/range)	3.50 (1.70–5.07)	3.50 (1.05–4.75)	0.51	3500 (1.70–5.07)	3.48 (1.05–4.75)	0.31	18/4.5
Maternal age at delivery, years (median/range)	29 (18–44)	28 (17–40)	0.13	29 (18–44)	28 (17–38)	0.12	11/2.8
Birth year	1993 (1991–1998)	1993 (1991–1998)	0.90	1993 (1991–1998)	1993 (1991–1998)		12/3.0
HLA-DQB1 alleles							
High risk *n* (%)	118 (59.3)	26 (13.9)		116 (64.1)	26 (16.9)		
Moderate risk	37 (18.6)	39 (20.3)		37 (20.4)	37 (24.0)		
Low or protective risk	28 (14.1)	104 (51.3)		28 (15.5)	91 (59.1)		45/11.3
missing	16 (8.0)	29 (14.4)	<0.001			<0.0001	

Comparison of cases and controls regarding birth characteristics and HLA-DQB1 genotypes. Only 188 controls are included in the third columns due to missing information regarding sex in 11 controls (Figure 1). Cases and controls were comparable for sex, gestational age, birth length, birth weight, and maternal age at delivery. Only HLA-DQB1 genotypes differed significantly between cases and controls. The grouping of HLA-DQB1 genotype: DQB1allele 1/DQB1allele 2: High risk: 03:02/99:99, 03:02/02, 06:04/03:02. Moderate risk: 06:04/99:99, 03:01/02, 02/99:99, 06:04/02, 06:03/03:02. Low/protective risk: 03:04/99:99, 06:02/03:02, 06:02/99:99, 06:02/02, 06:03/99:99, 03:01/99:99, 99:99/99:99, 06:02/03:01, 06:03/03:01, 06:04/03:01, 03:04/02, 06:03/02.

**Table 2:** The odds ratios for T1D development in newborns per doubling of iron content in blood.

Variables	Model 1		Model 2	
	OR (95% CI)	*p* Value	OR (95% CI)	*p*-Value
Iron content (doubling)	2.01 (1.04; 3.87)	0.032	2.55 (1.04; 6.24)	0.041
Other factors				
Sex				
Girls	1.04 (0.69; 1.55)	0.89	1.23 (0.72; 2.08)	0.45
Boys	1		1	
Gestational age (weeks)	0.94 (0.84; 1.06)	0.31	0.81 (0.68; 0.97)	0.022
Birth weight (100 g)	1.02 (0.98; 1.06)	0.34	1.04 (0.98; 1.10)	0.22
Maternal age (year)	1.03 (0.98; 1.08)	0.19	1.00 (0.95; 1.06)	0.90
HLA-DQB1 alleles				
High risk	17.2 (9.42; 31.3)		15.8 (8.38; 29.6)	
Moderate risk	3.44 (1.85; 6.39)		3.60 (1.88; 6.88)	
Low risk	1	<0.0001		0.0001

Model 1 is the test of each variable separately and the risk of type 1 diabetes (T1D) adjusted for season and birth year. Logistic regression is used to calculate the odds ratio (OR) with 95% confidence intervals (CI). Model 2 is the full model including all variables season, birth year, sex, HLA-DQB1 genotype, birth weight, gestational age, and maternal age at delivery. Birth length was omitted since birth weight and birth length were highly correlated. The grouping of HLA-DQB1 genotype: DQB1allele 1/DQB1allele 2: High risk: 03:02/99:99, 03:02/02, 06:04/03:02. Moderate risk: 06:04/99:99, 03:01/02, 02/99:99, 06:04/02, 06:03/03:02. Low risk: 03:04/99:99, 06:02/03:02, 06:02/99:99, 06:02/02, 06:03/99:99, 03:01/99:99, 99:99/99:99, 06:02/03:01, 06:03/03:01, 06:04/03:01, 03:04/02, 06:03/02.
